# Patient preferences in retinal drug delivery

**DOI:** 10.1038/s41598-021-98568-7

**Published:** 2021-09-23

**Authors:** Brandon Jacobs, Nicholas Palmer, Trupti Shetty, Helen Dimaras, Amir Hajrasouliha, Denis Jusufbegovic, Timothy W. Corson

**Affiliations:** 1 Eugene and Marilyn Glick Eye Institute and Department of Ophthalmology, Indiana University School of Medicine, 1160 West Michigan Street, Indianapolis, IN 46202 USA; 2 Hospital for Sick Children, Toronto, ON Canada; 3 Department of Ophthalmology and Vision Sciences, and Division of Clinical Public Health, Dalla Lana School of Public Health, Faculty of Medicine, University of Toronto, Toronto, ON Canada; 4 Present Address: Neurobiology, Neurodegeneration and Repair Laboratory, National Eye Institute, National Institutes of Health, Bethesda, MD USA

**Keywords:** Drug delivery, Translational research, Therapeutics, Quality of life, Retinal diseases

## Abstract

Retinal vascular diseases (RVDs) are often treated with intravitreally (IVT) injected drugs, with relatively low patient compliance and potential risks. Ongoing research explores alternative RVD treatments, including eye drops and oral tablets. This study surveyed RVD patients treated with IVT injections to establish factors influencing low compliance rates while gauging treatment delivery method preferences. Demographics, perspectives, and treatment preferences were collected via IRB-approved, self-administered survey sent to Glick Eye Institute patients treated via IVT injections. Demographics, diagnoses, and treatments were ascertained from respondents’ medical records. Gender, age, and number of IVT injections received were used as stratifications. Five-level Likert-style scales and t-tests evaluated responses and stratification comparisons. The most common diagnoses in the respondent population (n = 54; response rate = 5%) were age-related macular degeneration, macular edema, and diabetic retinopathy. Respondents had varying levels of education, income, and age. Most (83%) admitted feeling anxious prior to their first IVT injection, but 80% reported willingness to receive IVT injections indefinitely, with a preference for ophthalmologist visits every 1–3 months. Eye drops would be preferred over IVT injections by 76% of respondents, while 65% preferred oral tablets, due to several perceived negative factors of IVT injections and positive factors for eye drops. Stratified groups did not differ in responses to survey questions. RVD patients will accept IVT injections for vision preservation, but alternative delivery methods like eye drops or oral tablets would be preferred. Thus, development of eye drop and oral therapeutics for RVD treatment is further emphasized by these findings.

## Introduction

The current cornerstone of pharmacological treatments for retinal vascular diseases (RVDs) such as neovascular age-related macular degeneration (AMD) and diabetic macular edema is intravitreal (IVT) injections of anti-vascular endothelial growth factor (anti-VEGF) drugs^1,2^. Previous studies have found that lower compliance rates with IVT injection therapy are associated with clinic visit frequency, fear of the injections, lack of understanding of the chronic nature of neovascular diseases, and the COVID-19 pandemic^3–5^. Treatment non-compliance can worsen the overall prognosis of RVDs by breaking the necessary continuation of treatment required to keep neovascularization in check^6^. However, recent developments in small molecule anti-angiogenic drugs provide potential treatments for these RVDs that could utilize less invasive drug delivery systems, such as eye drops, oral tablets, and subconjunctival or suprachoroidal injections^7–10^. Non-invasive treatment options also avoid the rare but serious risks of adverse events with IVT injections, notably endophthalmitis^11^.

While eye drop preparations with regorafenib and pazopanib, which inhibit the VEGF receptors (VEGFR) and other kinases, have not proven clinically effective in humans^12^, oral VEGFR-1 and VEGFR-2 inhibitors have shown promise as therapeutics for neovascular AMD based on mouse model data^13–15^. Although tyrosine kinase inhibitors may result in systemic adverse effects^13^, VEGFR-1 and VEGFR-2 inhibitors are more precise in their ocular tissue uptake^14,15^. Multiple different VEGFR-2 inhibitors showed not only efficacy in animal models but also selective distribution to ocular tissue despite oral delivery^15^. Drugs not directly targeting VEGF are also a possibility, such as the Ref-1 inhibitor APX3330, effective as a systemic agent for choroidal neovascularization in mice and currently entering a Phase II trial as an oral tablet for diabetic macular edema (NCT04692688)^16^. One of many potential eyedrops is aganirsen, an antisense oligonucleotide targeting insulin receptor substrate 1, currently used for corneal neovascularization. It has been successfully used as an eyedrop to treat choroidal neovascularization in rabbits and non-human primates^17^.

Moving away from procedure-based treatments could allow for a more comfortable treatment for the patient and potentially fewer visits to the clinic. However, there are also provider concerns about fewer patient visits resulting in more challenging disease monitoring. While a balance must be struck between disease monitoring and patient fatigue of visits to the clinic, less invasive therapies could potentially increase patient compliance with treatment and thus increase treatment efficacy.

In order to determine how patients view IVT injection therapy as well as what patient preferences might be for future potential treatments, this study surveyed patients who had received IVT injection therapy. Most respondents were not comfortable with the idea of receiving IVT injections before their first treatment but were willing to continue treatment indefinitely to maintain their vision. When asked about future drug delivery options, most respondents reported that they would prefer either eye drops or oral tablets over IVT injections. The majority of respondents also reported that they would prefer eye drops over oral tablets.

## Methods

### Study design

This was a survey-based study covering respondents’ perspectives on treatment compliance, preferences, and socio-economic factors. Inclusion criteria were any adult patient who received IVT injection therapy at the Eugene and Marilyn Glick Eye Institute (Indianapolis, Indiana, USA) from January 2016 through July 2020. At this location, IVT injections are given on the same day of patients’ clinical visit, in the same room. No pre- or post-injection antibiotic eyedrops are given. Exclusion criteria were any person who had not received IVT injection therapy or did not provide consent to participate in the study or HIPAA authorization. A survey was sent by mail to eligible potential patients with options to complete the survey online (through a REDCap^18^ form) as well as have a large print version made available upon request. Informed consent was obtained in writing from subjects, after providing written explanation of the nature and possible consequences of the study. This study was conducted in accordance with the Declaration of Helsinki and was approved by the Indiana University Institutional Review Board.

### Respondents

For those who responded and provided consent, their age, gender, ethnicity, number of IVT injections, all IVT treatment drug(s) received, and all retinal clinical diagnoses were collected from their health records. Respondents’ views on IVT injection therapy and future treatments were collected through the study survey. Respondent information and survey responses were transferred to REDCap for data consolidation and security. For analysis, respondents were stratified by number of IVT injections received (< 4 vs. ≥ 4), by age (< 70 vs. ≥ 70), and by gender.

### Survey

The survey used in this study included Likert-style questions with possible responses of strongly disagree, disagree, neutral, agree, and strongly agree. These questions were grouped into categories focusing on what respondents thought about IVT injection therapy and how respondents would prefer to receive treatment if new delivery methods were made available. Sections of the survey focused on reasons for missed appointments, level of education, and preferred time between appointments used check boxes to indicate respondent responses. Other sections in the survey offered text entry fields for questions regarding respondent understanding of their retinal health, uncommon reasons for not attending appointments, and explanations as to why respondents prefer one drug delivery route over another. The survey was paired with information from respondents’ medical records to confirm documented ethnicity/race, gender, date of birth, residence, diagnoses, drugs received, and number of IVT injections received.

### Qualitative analysis

Written responses to the question, ‘Please explain why you ranked the treatments for retinal disease the way you did’ were evaluated using a qualitative descriptive method with grounded theory overtones^19^. Data were managed and coded using NVivo 12 QSR software. A qualitative content analysis was performed using an inductive approach, in which a coding frame was revealed by the data and adjusted as the analysis progressed. Using constant comparative methods, responses were compared and contrasted, and categories were grouped and regrouped to represent higher levels of abstraction that revealed main themes.

### Statistical analysis

Numerical values were assigned to respondent responses for Likert-style questions, with a value of one being assigned to a response of ‘strongly disagree’ through to a value of five for ‘strongly agree’. The mean scores between subgroups were statistically evaluated using Student’s t-test^20^ and median and mode also reported. Questions in the survey that were not Likert-style questions (i.e., the yes or no questions) were evaluated by frequency of respondent responses.

## Results

### Respondent demographics

One thousand and fifty patients fit the inclusion criteria. A total of 68 responses were received (60 by mail, 8 electronically), and 14 responses were excluded for incomplete HIPAA authorization and/or informed consent forms. Respondents (n = 54; response rate = 5%; Table 1) were well-diversified in levels of education, income, and age ranging from 27 to 91 years. Documented RVDs among these respondents included AMD, diabetic retinopathy, macular edema, and others (Table 1). All pertinent ocular diseases for each patient were documented even if they were not the primary reason for IVT treatment. Except where indicated below, there were no significant differences in question responses between age (< 70 years), gender, or number of IVT injection (< 4 injections) stratifications.

**Table 1:** Demographics of study respondents. n = 54.

Gender	n (%)
Male	22 (41%)
Female	27 (50%)
Unspecified^a^	5 (9%)
**Race/ethnicity**	
White	37 (69%)
Non-white	5 (9%)
Unspecified^a^	12 (22%)
**Education level**	
Less than high school diploma	1 (2%)
High school diploma or equivalent	17 (31%)
Associate’s degree	7 (13%)
Bachelor’s degree	12 (22%)
Graduate school	16 (30%)
Prefer not to answer	1 (22%)
**Household income**	
Less than $20,000	14 (26%)
$20,000–34,999	9 (17%)
$35,000–49,999	7 (13%)
$50,000–74,999	6 (11%)
$75,000–99,999	6 (11%)
Over $100,000	7 (13%)
Prefer not to answer	5 (9%)
**Documented retinal diagnoses**^**b**^	
Age-related macular degeneration	14 (26%)
Diabetic retinopathy	18 (33%)
Retinal vein occlusion	10 (19%)
Retinal detachment	3 (6%)
Macular edema	21 (39%)
Glaucoma	3 (6%)
Other	13 (24%)
**Treatment drug(s)**^**c**^	
Aflibercept	21 (39%)
Bevacizumab	34 (63%)
Ranibizumab	3 (6%)
Other	7 (12%)

^a^Unspecified due to lack of full medical record documentation.

^b^Some respondents had more than one diagnosis; all ocular diagnoses were recorded even if they were not the primary reason for IVT injection treatment.

^c^Some respondents were treated with multiple drugs over the course of their disease(s).

### Opinions on IVT injections

**Figure 1:**
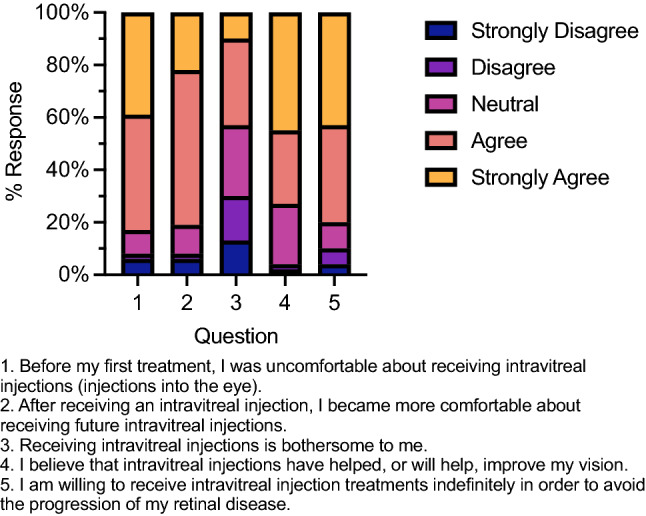
Opinions on IVT therapy expressed as a 100% stacked column chart of Likert-scaled responses from survey questions. n = 52–54 responses as in Table 2.

Before their first IVT injection treatment, 83% of respondents ‘agreed’ or ‘strongly agreed’ to feeling apprehension about the injection (Fig. 1); Table 2 gives the median and mode for each response. This apprehension appears to dissipate as reported by about 82% of respondents who answered ‘agree’ or ‘strongly agree’ and shown by the median and mode ‘agree’ response correlating to feeling more comfortable after the first injection. Furthermore, a median response of ‘agree’ and mode response of ‘strongly agree’ were found regarding respondents’ belief that IVT therapy will improve their overall disease progression with 74% answering positively (‘agree’ or ‘strongly agree’) to this question. Median ‘agree’ and mode ‘strongly agree’ responses related to the respondents’ willingness to receive IVTs indefinitely to preserve their vision. Regardless, the median ‘neutral’ and mode ‘agree’ responses suggest that IVT injections may still be considered bothersome to some respondents with a positive response in about 42% of respondents and a negative response (‘disagree’ or ‘strongly disagree’) in 31%.

**Table 2:** Response to Likert-scaled survey questions about intravitreal (IVT) injections, other potential non-compliance factors, and alternative treatment methods.

Question	Median	Mode
Apprehension before 1st IVT (n = 54)	Agree	Agree
More comfortable after 1st IVT (n = 54)	Agree	Agree
IVT is bothersome (n = 52)	Neutral	Agree
Believe IVT will help (n = 53)	Agree	Strongly agree
Willing to receive IVT indefinitely (n = 54)	Agree	Strongly agree
Preferred clinic visit interval (n = 53)	3 months	1 month
Transportation not difficult (n = 53)	Agree	Strongly agree
Clinic too far away (n = 54)	Disagree	Strongly disagree
Have reliable partner to attend with (n = 53)	Agree	Agree
Concerned about coverage & co-pay (n = 53)	Neutral	Agree
Prefer eye drops over IVTs (n = 52)	Agree	Strongly agree
Eye drops are difficult to use (n = 51)	Disagree	Disagree
Prefer oral tablets over IVTs (n = 52)	Agree	Agree
Oral tablets are difficult to use (n = 52)	Strongly disagree	Strongly disagree

### Other potential non-compliance factors

**Figure 2:**
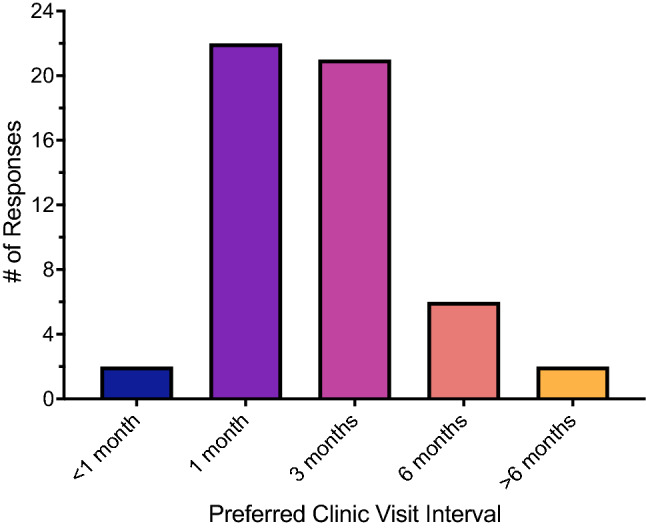
Opinions on preferred visit interval expressed as a histogram of survey responses. n = 53 responses as in Table 2.

**Figure 3:**
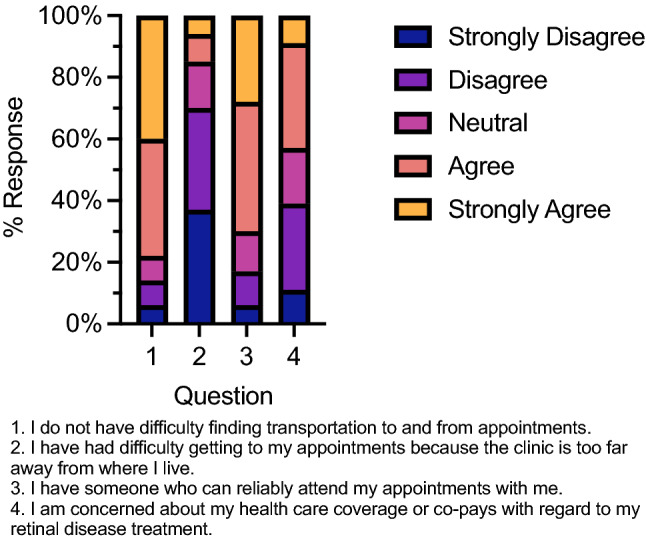
Responses from survey questions exploring potential factors that may influence patient non-compliance, expressed as a 100% stacked column chart of Likert-scaled results. n = 53–54 responses as in Table 2.

Other potential factors affecting non-compliance were also explored (Figs. 2, 3; Table 2). About 41% of respondents preferred monthly clinic visits while 39.6% preferred visits spaced out every 3 months (Fig. 2). The median response was 3 months while mode was 1 month; few responses were seen in the other interval options (< 1 month, 6 months, and > 6 months).

About 77% of respondents claimed no difficulties with transportation to the clinic with a median ‘agree’ and mode ‘strongly agree’ response to this question (Fig. 3). Likewise, clinics being too far away received a median ‘disagree’ and mode ‘strongly disagree’ response, corresponding to an overall 70% of respondents refuting this possible factor. About 70% of respondents also reported having a reliable partner to attend clinic visits with them with both median and mode responses of ‘agree’. When asked about concerns for healthcare coverage and insurance co-pays, about 43% of respondents responded positively to this concern while 40% reported disagreement or strong disagreement with this factor. The median response for healthcare concern was ‘neutral’ while its mode was ‘agree’. Stratifying by age revealed a significantly higher concern for healthcare coverage and co-pays (*p* = 0.026) in the older (> 70 years of age; n = 24; mean = 3.5) subgroup compared to the younger subgroup (≤ 70; n = 24; mean = 2.7). Health insurance coverage information was not collected, so further analysis within this demographic could not be conducted.

Despite these findings, about 36% of respondents admitted to missing at least one appointment. Reasons included fear of IVT injection, believing treatment would not help or was not needed to help their vision, being too busy at the time, transportation conflicts, financial conflicts, physical inability, switching clinics, and COVID-19 pandemic-related cancellations (Table 3).

**Table 3:** Reported reasons for missing appointments. Some respondents chose more than one reason. n = 19.

Reason	n (%)
Physically unable to attend	5 (26%)
Transportation problems	4 (21%)
Fear of injection	2 (11%)
Did not feel IVT was needed	2 (11%)
Too busy	2 (11%)
Financial limitations	2 (11%)
Began receiving care at another clinic	2 (11%)
COVID-19 pandemic-related cancellation	2 (11%)
Believe IVT was not beneficial	1 (5%)
Other	3 (16%)

### Opinions on alternative treatment delivery methods

**Figure 4:**
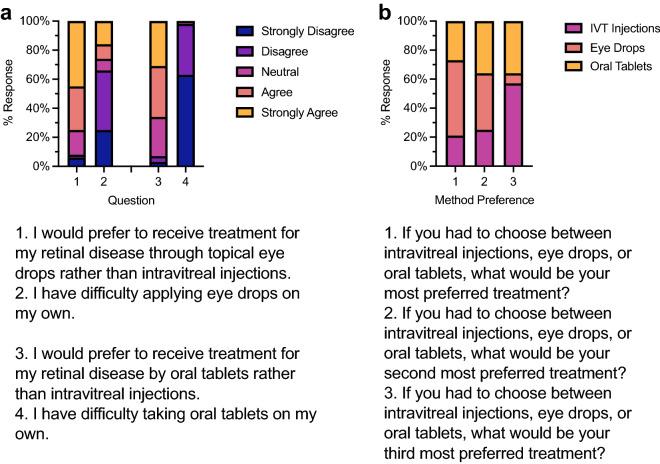
(**a**) Opinions on alternative treatment delivery routes to IVT injections expressed as a 100% stacked column chart of Likert-scaled responses from survey questions. n = 51–52 responses as in Table 2. (**b**) Frequency of preferred treatment delivery method by rank (1 = most preferred; 3 = least preferred). 1st choice n = 52; 2nd choice n = 44; 3rd choice n = 44.

We found that there is a strong preference to alternatives to IVT therapy for RVDs (Fig. 4a; Table 2). About 76% of respondents claimed they would prefer eye drops over IVT injections, and 65% would prefer oral tablets over IVT injections. The median for eye drops preferred was ‘agree’ while its mode was ‘strongly agree,’ and oral tablets preference had both median and mode of ‘agree’. Male respondents (n = 22; mean = 3.6) had significantly lower preference for eye drop therapy (*p* = 0.009) than female respondents (n = 26; mean = 4.5).

About 67% of respondents claimed to not have difficulty self-administering eye drops with both a median and mode response of ‘disagree’ to such difficulties. Likewise, both a median and mode response of ‘strongly disagree’ were reported when asked of difficulties taking oral tablets. Those who responded ‘strongly disagree’ or ‘disagree’ to this question made up 98% of respondents.

Respondents ranked IVT injections, oral tablets, and eye drops. A majority (52%) of respondents reported preferring eye drops as a potential first choice for treatment, making eye drops a clear first choice preference (Fig. 4b; Table 2). Interestingly, oral tablets had a fairly even distribution among each of the three ranks. Lastly, IVTs achieved a 57% response rate for the third choice, making it the least desirable therapy of the three options presented.

### Qualitative analysis of treatment delivery preferences

Of 54 respondents, 48 (89%) provided narrative responses explaining their ranking of the three treatment methods. Responses were categorized into three main themes: (1) internal factors influencing respondent preferences, further sub-categorized into perceived positives and negatives of each treatment method; (2) external factors influencing respondent preferences; and (3) acceptability of IVT injections (Table 4).

**Table 4:** Reported reasons for future treatment delivery preferences.

Category	Sub-category	Theme	n	Example quote
Internal factors influencing patient preferences	Positives: eye drops	Ease of use	5	Eye drops are easy to apply
		Comfortable	5	Eye drops would be most comfortable
		Self-administration	2	I could take the eye drops or tablets on my own
		Effect	1	Eye drops might be more effective than tablets
	Positives: oral tablets	Ease of use	7	Taking an oral medication is the easiest
		Self-administration	3	can be done at home without a trip to facility
	Positives: IVT	Effect	5	I think injections would work faster and better
	Negatives: IVT	Physical burden (pain, discomfort, interference in daily activities)	7	injections are…painful and it’s hard to drive or work afterwards
		Complicated (require office visits; dangerous; more involved)	5	I feel injections can be dangerous but have no other option
		Emotional burden (stress, fear)	4	an injection in the eye is REALLY HORRIFYING
		Modest effect	1	For the modest benefit from injections, I would prefer eye drops
	Negatives: oral tablets	Side effects	6	Oral worries me for systemic side effects
		Uncertain effect	2	I doubt oral tablets would be effective
		Difficult	3	Oral tablets would be easy but I take a lot of meds and hate to add one more
External factors influencing patient preferences	Effect		4	I would want the treatment which works the best
	Medical advice		1	I am willing to take treatment in any form and would follow the doctor's best advice
Acceptability of IVT			4	The injections were uncomfortable compared to the other 2 possibilities, but I had great success so happy to continue if necessary

Some respondents spoke to more than one theme. n = 48 narrative responses.

*Internal factors* were defined as personal thoughts, experiences or views about the treatment methods which appeared to influence preferences. Perceived positives were indicated for all treatment modalities. Eye drops were preferred because they were perceived to be (1) easy; (2) comfortable; (3) able to be self-administered; and (4) effective. Oral tablets were preferred because they were perceived to be (1) easy; and (2) able to be self-administered. Those who preferred IVT injections explained that this was because of their perceived effect to treat their condition. Perceived negatives were indicated only for oral tablets and IVT injections. The main reasons oral tablets were not preferred were perceptions they were (1) associated with side effects (either alone or in combination with other medications), (2) difficult to use, and (3) not as effective. IVT injections were not preferred because they were considered to be (1) associated with physical burden, such as pain, discomfort or interference in daily activities; (2) complicated (e.g., requires clinic visits, dangerous, more involved); (3) emotionally burdensome, such as inducing stress or fear; and (4) modestly effective.

*External factors* were defined as information or views beyond the respondent themselves which influenced their preferences. These fell into two categories: (1) effect (i.e., the respondent prefers the treatment that works best) and (2) medical advice (i.e., the respondent prefers the treatment that the physician suggests).

*Acceptability* of IVT injections: even among those who did not prefer IVT injections, several indicated that the mode of therapy was still acceptable. Acceptability of IVT injection as a mode of treatment was mainly due to its perceived efficacy and it being necessary for treatment of their condition at this time.

## Discussion

### Summary

Our results suggest that the majority of respondents feel apprehensive prior to their first IVT injection, and while this apprehension may fade after their first treatment, IVT injections are still considered bothersome to many of them. Despite this, most respondents are still willing to receive IVT therapy indefinitely if it means adequate preservation of their vision. Furthermore, respondents did not report difficulties with multiple aspects of clinic access. However, an overall concern for healthcare coverage and co-pays by respondents was identified as a potential factor affecting non-compliance, especially in the upper age range of RVD respondents.

Strong preference toward alternative delivery options of eye drops and oral tablets over IVT injections for treatment of RVDs was reported, with minimal reported difficulties with self-administration of these alternative methods. This notion may suggest an increase in compliance if these therapeutic choices were available. However, respondents’ free response explanations to their preferences often indicated a desire for whatever would work best rather than assuming equal efficacy across each delivery method proposed. A clinic visit interval preference strongly favoring visits every 1–3 months suggests respondents’ concern for their visual health that may allow for continued, regular visits for clinicians to monitor ongoing disease progression, even if the visits were not necessary for physician-administered IVT injections.

### IVT therapeutics

This survey revealed respondents’ overall wariness toward IVT injections. Anti-VEGF IVT therapy results in significantly better outcomes for RVDs, such as neovascular AMD and diabetic retinopathy, compared to the previous gold standard, panretinal photocoagulation^21,22^. Despite this therapeutic efficacy, low compliance rates reduce potential positive outcomes in real-world scenarios. A US cohort study of 9007 patients undergoing IVT injection treatment for neovascular AMD found a loss to follow-up rate of 22% with nearly all of these patients not attending a rescheduled visit within 12 months. This study also exhibited comparatively higher losses to follow-up among several minority groups, and with increasing age, lower adjusted income, and greater distance to clinics^23^. Furthermore, a Turkish study found that nearly 40% of neovascular AMD patients treated with IVT ranibizumab failed to fully comply with pro re nata treatment by their 1-year mark, citing several factors that affected their observed non-compliance rates, such as fear of injection, socioeconomic status, education level, and disbelief of treatment efficacy as major influences upon phone interview follow-up^5^. Similar poor compliance rates are seen with IVT treatments for diabetic macular edema, as well; missed treatments were closely related to loss of visual acuity in this disease^24,25^. Similarly, our survey uncovered that 36% of respondents admitted to missing at least one appointment throughout their treatment, citing similar factors of fear, disbelief, and finances along with other factors described above. However, our survey did not specify missed appointments as only being missed injection treatments. Missed appointments and dissatisfaction with IVT injection treatment could be even higher in regions where separate surgical visits are required to receive injections, rather than same-day office visits as done at our site.

Furthermore, an estimated 20–30% of RVD patients may not even agree to initiate treatment, and therefore, would not be considered in non-compliance analyses^26^. Additionally, under-prescription of IVT injections may also affect real-world applications of this treatment method. Since IVT injection therapy is typically prescribed on a pro re nata basis, it may not be achieving the same functional outcomes as primary clinical trials had shown. Under-prescription is influenced by similar factors to non-compliance such as socioeconomic status, transportation, and access to other resources^25,27^. For IVT therapy, there are no precise definitions for non-compliance, non-persistence, or non-adherence which may cause biases when assessing these rates in the literature^27^. Thus, despite the efficacy of IVT therapeutics, poor persistence and compliance rates may continue to hinder the overall number of positive outcomes among affected patients.

In addition to compliance issues, IVT injections carry risks of complications which may at least partly explain the apprehension reported by this survey. Endophthalmitis, retinal detachment, increased intraocular pressure, intraocular hemorrhage, lens pathology, and many others in addition to some rarer systemic effects including anaphylaxis and cardiovascular events, such as myocardial infarction and stroke, have been linked to IVT injections^11^. While there are no actively prescribed oral or topical therapeutics to compare with these adverse effect rates, it is likely that the majority of directly ocular-related complications would be lower due to the far less invasive nature of topical and oral drug delivery methods. However, systemic effects that are fairly rare with IVT injections may be increased in both oral and eye drop therapeutics requiring higher drug concentrations to overcome the various biological barriers each delivery method is challenged with^28^.

Another consideration for increasing rates of non-compliance is events such as the ongoing SARS-CoV-2 pandemic. Two of our survey respondents listed ‘COVID’ as the reason for missing an IVT injection. An estimated 79% initial decrease in ophthalmology clinical visits in the United States in April 2020 occurred due to pandemic-related reasons. While this decrease was less severe during the subsequent month, a cumulative decrease of 47% in ophthalmology clinical visits was estimated as of June 2020^29^. Given the standard frequency of clinic visits for RVD patients, this decrease in visits may have resulted in reductions in patients’ visual acuity. Another study supported this notion after comparing the number of clinic visits and IVT injection treatments fulfilled in the same 4-week span, from March 15 to April 14, across the last five consecutive years including 2020 at a site in Israel. They found about a 50% decrease in the number of IVT injection treatments in the 4-week span from the year 2020 compared to the averages of the previous four years^3^. Adding this pandemic factor into the various other non-compliance risks only exacerbates the potential for overall poor outcome rates associated with the IVT mode of treatment despite their drugs’ proven efficacies.

### Topical ocular therapeutics

This survey revealed a strong respondent preference towards potential eye drop therapeutics instead of IVT injections. Unfortunately, topical therapeutics in the form of eye drops have not yet been approved for RVDs. Nonetheless, this is an active area of research, especially with enhancement of topical ocular drug delivery^30^.

However, eye drop compliance is another important consideration, despite respondents’ self-reported preference for this method in our survey; no perceived negatives to this route were noted by our respondents. Two previous studies found that only about 60% of patients properly completed eye drop regimens while the remaining patients had missed doses^31,32^. In one study, only about 62% of patients administered their own drops, and only 9 of 30 patients successfully aimed an eye drop at a target^31^. Other issues noted by these studies included arthritis or other problems in patients making it difficult to administer drops, expelling more than one drop at a time, and contacting the dropper tip directly to the eye^31,32^. Similarly, another study demonstrated that over 50% of glaucoma patients exhibited non-compliance and/or improper administration technique^33^. Respondents to our survey may not have considered these difficulties with proper eye drop administration techniques upon answering. Nonetheless, aids such as eye drop compliance charts^34^, Medication Event Monitoring System eye droppers^35^, and other patient education interventions may be developed in the future to further increase eye drop compliance in accordance with satisfying patient preferences of therapy choice as discovered by our study.

### Oral therapeutics

Our survey respondents indicated a secondary preference for oral therapy. However, for the treatment of RVDs, oral therapeutics may face efficacy roadblocks including the blood-retina barrier and increased risk of systemic toxicity^36^. The risks of both systemic side effects and limited efficacy were acknowledged by our respondents. Despite these challenges, some oral therapeutics for retinal vascular disease are undergoing active research. Our findings here suggest that higher compliance rates compared to IVT injections for treatment of RVDs may be achievable through oral drug delivery, given its preference among our respondents. AREDS Report No. 7 reported that about 79% of patients studied had taken at least 75% of their oral tablet regimen for dry AMD after 5 years of follow-up^37^. On the other hand, older patients, who make up a significant portion of the RVD population, are likely taking multiple other oral medications which may cause issues related to polypharmacy. Some respondents to our survey explained their preference against oral tablets to avoid adding another pill to their regimen.

### Limitations

A major limitation is this survey’s low response rate and consequently small sample size. Although the education level, income range, and age demographics of respondents appear to have a broad range, our racial diversity representation is lacking with very few non-white respondents. Unfortunately, this disparity is a common limitation among such studies. Also, the included respondents were all residents of one American state (Indiana) and most respondents were residents of Marion County (Indianapolis). Additionally, as in most survey studies, respondent bias may have influenced the results since those who feel strongly about the topic are more likely to respond and voice their opinions. Therefore, generalizing these findings to the broader population should be done with caution.

Furthermore, some verbiage may not have been clear enough for respondents to respond in a non-biased way. For instance, it is unclear whether the significant response claiming ideal clinic visit frequency of 1–3 months was due to respondents’ familiarity with that time frame as explained by their physicians or an actual desire to keep up with their ocular health on a frequent basis. Likewise, when ranking treatment preferences, some respondents only checked their most desirable treatment method rather than ranking all of them 1–3. Given the strong differences between these preferences overall, it is unlikely the results were severely affected by any misinterpretations. To keep the survey manageable, we also did not explore other potential delivery methods such as implants, suprachoroidal or subconjunctival injections. These shortcomings warrant further studies to confirm and expand these findings with a larger sample that includes more minority representation.

## Conclusion

A majority of survey respondents with RVDs reported that they are willing to receive IVT injections indefinitely in order to preserve their vision. However, many still consider IVT therapy to be bothersome, and most respondents reported preferences to alternative drug delivery routes such as eye drops or oral tablets over IVT injections if those treatments were available. Furthermore, respondents claimed an ideal clinic visit interval between one and three months which should allow for regular disease progression monitoring by clinicians regardless of therapy regimen. These findings underscore the value of continuing to develop new therapeutics with alternative delivery routes that are more in line with patient preferences. Less invasive treatments could increase patient comfort and open up opportunities for prescription-based treatments as opposed to the clinic visits required for each IVT injection. Higher patient comfort and more accessibility of treatment could aid in advancing patient compliance with RVD treatment, increasing treatment efficacy overall.

## Data Availability

Deidentified data underlying the figures and tables are available from the authors on reasonable request.
